# Intestinal phages interact with bacteria and are involved in human diseases

**DOI:** 10.1080/19490976.2022.2113717

**Published:** 2022-08-29

**Authors:** Han Shuwen, Ding Kefeng

**Affiliations:** a Department of Colorectal Surgery and Oncology, Key Laboratory of Cancer Prevention and Intervention, Ministry of Education, The Second Affiliated Hospital, Zhejiang University School of Medicine, Hangzhou, China; b Department of Medical Oncology, Huzhou Central Hospital, Huzhou, China; c Department of Colorectal Surgery and Oncology, Cancer Center Zhejiang University, Hangzhou, China

**Keywords:** Intestinal microbiota, phage, bacteria, phage-related diseases, phage therapy

## Abstract

Phages are the most abundant and diverse biological entities on Earth and exert specific effects on bacterial hosts. The coexistence of phages and bacteria in the intestinal tract is dynamic and interdependent. Phages are involved in maintaining the stability and composition of the bacterial community, and an imbalance in phages and bacteria in the intestinal tract can cause diseases. This review elucidates interactions between phages and bacteria in the human intestinal tract and their roles in the pathogenesis and treatment of diseases. Understanding the relationship among phages, bacteria and host diseases is conducive to promoting the application of phages in the treatment of human diseases.

**List of abbreviations:**

EMBL-EBI The European Bioinformatics Institute; E. coli Escherichia coli; E. faecalis Enterobacter faecalis; B. fragilis Bacteroides fragilis; B. vulgatus Bacteroides vulgatus; SaPIs Staphylococcus aureus pathogenicity islands; ARGs Antibiotic resistance genes; STEC Shiga toxigenic E. coli; Stx Shiga toxin; BLAST Basic Local Alignment Search Tool; TSST-1 Toxic shock toxin 1; RBPs Receptor-binding proteins; LPS lipopolysaccharide; OMVs Outer membrane vesicles; PT Phosphorothioate; BREX Bacteriophage exclusion; OCR Overcome classical restriction; Pgl Phage growth limitation; DISARM Defense island system associated with restrictionmodification; R-M system Restriction-modification system; BREX system Bacteriophage exclusion system; CRISPR Clustered regularly interspaced short palindromic repeats; Cas CRISPR-associated; PAMs Prospacer adjacent motifs; crRNA CRISPR RNA; SIE; OMPs; Superinfection exclusion; Outer membrane proteins; Abi Abortive infection; TA Toxin-antitoxin; TLR Toll-like receptor; APCs Antigen-presenting cells; DSS Dextran sulfate sodium; IELs Intraepithelial lymphocytes; FMT Fecal microbiota transfer; IFN-γ Interferon-gamma; IBD Inflammatory bowel disease; AgNPs Silver nanoparticles; MDSC Myeloid-derived suppressor cell; CRC Colorectal cancer; VLPs Virus-like particles; TMP Tape measure protein; PSMB4 Proteasome subunit beta type-4; ALD Alcohol-related liver disease; GVHD Graft-versus-host disease; ROS Reactive oxygen species; RA Rheumatoid arthritis; CCP Cyclic citrullinated protein; AMGs Accessory metabolic genes; T1DM Type 1 diabetes mellitus; T2DM Type 2 diabetes mellitus; SCFAs Short-chain fatty acids; GLP-1 Glucagon-like peptide-1; A. baumannii Acinetobacter baumannii; CpG Deoxycytidylinate-phosphodeoxyguanosine; PEG Polyethylene glycol; MetS Metabolic syndrome; OprM Outer membrane porin M.

## 1. Introduction

The human body is a complex symbiotic organism composed of its own cells and a large number of symbiotic microorganisms. Human microecology includes intestinal microecology, skin microecology, respiratory tract microecology, etc. However, intestinal microecology is the most important in the human microecosystem. Intestinal microecology imbalance may induce systemic diseases.^1^ Eckburg PB et al. detected 13,355 prokaryotic ribosomal RNA gene sequences in multiple parts of the colonic mucosa and feces of healthy individuals and found that there were at least 1,000 known bacteria in the intestinal tract of each individual. Ninety-eight percent of intestinal bacteria can be classified as *Bacteroidetes, Firmicutes, Proteobacteria* and *actinomycetes*. Most of the remaining bacteria have yet to be identified, corresponding to uncultured species and unrecognized microorganisms.^2^ In addition, there are thousands of viruses in the intestinal tract. By analyzing more than 28,000 intestinal microbiome samples and 2,898 reference genomes of cultured gut bacteria collected in various parts of the world, researchers from the Wellcome Sanger Institute and the European Bioinformatics Institute (EMBL-EBI) have identified more than 140,000 nonredundant gut phage genomes in the human intestinal tract.^3^ Liang G et al. and Carding SR et al. suggested that phage‒bacterial ratios were basically maintained at 1:1 in the human gut.^4,5^

Phages are the most common viruses in the human intestine (up to 10^8^ virus-like particles (VLPs) per milliliter in fecal filtrate).^6^ A special attribute of phages is that they are hosted exclusively in bacteria. Phages were first discovered in *Staphylococcus* and *Shigella* in 1915, and the best-known among them today is phage T4, with *Escherichia coli* serving as its host.^7^ Phages share similar characteristics with other types of viruses in the fact that they have small sizes (taking *E. coli* T4 phage as an example, its head size is approximately 95 × 65 nm, its tail is 95 ~ 125 nm long and its tubular structure has a 13 ~ 20 nm diameter), no complete cellular structures and a single nucleic acid segment. Based on their life cycle, phages can be classified into lysogenic phages^8^ and lytic phages.^9^ Lysogenic phages, also known as temperate phages, integrate their viral genetic material into the genome of the host bacteria, where their DNA or RNA is replicated by bacterial chromosomes through normal cell division without cell lysis/cleavage.^10^ In contrast, lytic phages, whose replication cycle is relatively complex, result in host bacteria lysing following four processes: adsorption, osmosis, synthesis, and release.^11^ Based on the frequency of their presence in the human intestine, phages can be further classified into low-compatibility phages, conventional phages, and core phages, which are present in 2–19%, 20%-50%, and more than 50% of the human population, respectively.^11^ The most widely distributed intestinal phage found in recent studies is a type that can infect *Bacteroidetes*, called “crAssphage”.^12^

It is typical that phages interact specifically with a single strain of bacteria. Phage–bacterial interaction networks are nested and modular. These interactions are continuously evolving, although their evolution may be influenced by localization in organs and tissues and the complexity of the interaction network.^13^ Phage activity affects the number and behavior of host bacteria and mediates gene transfer between bacteria during host inflammation. Phages are related to microecological balance and imbalance, and they can affect human health via predation of the bacterial ecological landscape or via more indirect routes, such as influencing metabolism and the immune system. Regulating the relationship between phages and bacteria can maintain the health of the body and even reverse diseases. In the 1930s, phages were used to fight infection in the United States, and later large-scale successful clinical trials were conducted on their use.^14^ Subsequently, phages have been widely used for the prevention and treatment of *bacillary dysentery* and *staphylococcal* infection in South America.^15,16^ In recent years, phages have been widely used for ultrasensitive biomarker detection, enhanced biological imaging for disease diagnosis, targeted drugs and gene delivery, effective vaccination, replacement of antibiotics for sterilization, and more.

This paper discusses in detail the mode of phage action on bacteria, the mode of bacterial rejection of phages, and the coevolution of phages and bacteria in the human intestinal tract. The relationship between phages and host diseases and recent applications in the medical field are further discussed. Investigating the potential of phage therapy may provide a reference for phages as antimicrobial agents.

## 2. The relationship between phages and bacteria in the intestine

### 2.1 Effects of bacteriophage invasion on the intestinal flora

## (I) phage-specific lysis-susceptible bacteria

The *Escherichia* virus PDX is a member of the strictly lytic *Myoviridae* family. It was reported that *Myoviridae* phage PDX killed a disease-associated enteroaggregative *E. coli* (EAEC) isolated from a child from rural Tennessee and an EAEC isolated from a child from Columbia in a dose-dependent manner. Cepko LCS et al. further found that EAEC reduced the β-diversity of the human microbiota, while *Myoviridae* phage PDX could kill EAEC without causing dysregulation of the human microbiome.^17^ Lytic phages were injected into conventional mice colonized with a group of identified human symbiotic bacteria. Longitudinal tracking of each microbial response using high-throughput sequencing and quantitative PCR showed that phages T4, F1, B40-8, and VD13 lysed only their susceptible bacteria *E. coli, Clostridium sporogenes, Bacteroides fragilis* and *Enterococcus faecalis*, respectively. These phages showed no significant effect on other symbiotic bacteria.^18^ An in vitro small intestine model was used to analyze the effects of a DSM 1058 phage preparation on preselected target *E. coli* strains and nontarget bacterial populations. It was found that the phage preparation of *E. coli* DSM 1058 affected only the population number of *E. coli*. However, other “symbiotic” bacterial species included in the intestinal model, such as *Streptococcus salivarius, Streptococcus lutetiensis* and *E. faecalis*, were not affected.^19^

## (II) phages affect the intestinal flora through horizontal gene transfer

Phages can significantly shape the ecosystem structure by strain-specific predation and mediate horizontal gene transfer by lysing host bacteria.^20,21^ Phages that can package the DNA of a bacterial host and transfer it to a new host are often called transduction phages.^22^ *Staphylococcus aureus*’s highly mobile toxin-carrying pathogenic islands (SaPIs) are particularly suited for packaging and transfer by specific staphylococcal phages. SaPI-encoded toxic shock toxin 1 (TSST-1) and other superantigens are inserted into specific chromosomal sites, where they are excised and replicated by temperate phages. After replication, SaPI DNA is packaged into special small infectious particles that produce specific transfer.^23,24^ By monitoring the transduction of a sodCIII::neo cassette (a gene sequence) in the Fels-1 prophage from LT2 to a recipient Salmonella strain, Bearson BL et al. confirmed that carbadox induced phage-mediated gene transfer.^25^ In multidrug-resistant strains DT104 and DT120, carbadox induced generalized transduction of phages, resulting in transfer of chromosome and plasmid DNA containing antibiotic resistance genes (ARGs).^25,26^ Modi et al. established mouse models of ciprofloxacin and ampicillin resistance and detected phage in feces. They found that phages in feces from ciprofloxacin-resistant mice carried genes encoding quinolone efflux pumps (e.g., NorM, mexD, and mexF), while phages in ampicillin-resistant mice carried genes encoding sensors and response regulators for cell wall synthesis inhibitors (e.g., VanRS) (3-fold increase in reads). The results indicated that phage could regulate the drug resistance of antibiotic-treated bacteria by encoding antibiotic resistance genes.^27^ By reanalyzing data from Modi et al.’s paper, Enault et al. found that there was also a two- to threefold increase in the bacterial-only clusters of orthologous groups of proteins (COGs). All kinds of bacterial genes were detected more frequently in the microbial metagenome of mice treated with ciprofloxacin and ampicillin, and there was no special selection for ARGs.^28^ This might be due to protophage induction by antibiotic treatment, with some subpopulations of protophage performing generalized transduction. Antibiotic treatment expanded the interaction between phages and bacterial species, leading to more tightly connected gene exchange networks between phages and bacteria.^27,28^

## (III) phages encode virulence factors of the bacterial population

Phages can spread virulence factors between strains, including toxin-coding genes that cause many diseases, such as diphtheria, cholera, dysentery, and scarlet fever.^29^ *Vibrio cholerae*, the pathogen of cholera, requires two coordinating regulators to achieve full virulence: cholera toxin and toxin-coregulated pilus. The structural genes of cholera toxin are encoded by the filamentous phage *CTXφ*, and the *CTXφ* genome acts as a plasmid for chromosome integration or replication. The El Tor mutation of the phage *CTXφ* destroys XerC and XerD, two bacterial-encoded tyrosine recombinases. These two enzymes usually play a role in the decomposition of chromosomal dimers. *CTXφ* phages integrate at the decomposition site DIF1 of the larger dimer of the two chromosomes of *V. cholerae*, leading to the genetic diversity of cholera epidemic strains and further affecting the release of cholera toxin by *V. cholera*e.^30,31^ The toxin in Shiga toxigenic *E. coli* (STEC) is encoded by resident temperate lambdoid bacteriophages. Temperate lambdoid bacteriophages might contain toxin structural genes or regulators of toxin structural genes transduced by host bacteria. The Shiga toxin (Stx) gene is expressed when the phage is induced to leave its dormant state and begin replication. Extensive phage replication results in the release of large amounts of Stx from *E. col*i.^32,33^ The Stx gene is located downstream of the phage PR promoter, and the transcription of the promoter and the expression of Stx are controlled by the Q antitermination protein. Q antitermination protein is expressed only during phage lysis-mediated growth, so phages carrying the Q21 subtype produce a lower amount of Stx.^32^

## (IV) phages involved in the regulation of bacterial metabolism

Specific predation by phages targeting bacterial species may eliminate the production of relevant bacterial metabolites. The relative abundance of different phages and bacterial metabolites in the intestinal tract of severely depressed patients and healthy persons has been monitored, and a symbiotic network has been thus constructed, showing that the abundance of *Klebsiella* phage (vB KpnP SU552A) was positively correlated with *Bacteroidetes* abundance and negatively correlated with proline, cysteine and tryptophan levels, affecting amino acid metabolism.^34^ Tryptamine is a neurotransmitter produced by the decarboxylation of tryptophan in a few symbiotic intestinal bacteria. Tryptophan decarboxylase-encoding genes have been identified in species such as *Ruminococcus gnavus* and *C. sporogene*s.^35^ Hsu BB et al. first used the Basic Local Alignment Search Tool (BLAST) to search tryptophan decarboxylase amino acid sequences from *C. sporogenes* (clospo_02083) against the other members of their consortium. The results showed that the amino acid sequence of the tryptophan decarboxylase from *C. sporogenes* (clospo_02083) had poor protein homology (top hit: 31% identity) with the other members of the consortium. The amino acid sequence of the tryptophan decarboxylase from *C. sporogene*s maintained its unique association with *C. sporogenes*. Then, the researchers collected fecal samples from germ-free mice colonized by the defined bacterial consortium and treated them with T4 and F1 phages. During treatment with the T4 and F1 phages, the researchers detected a 10-, 17-, and 2-fold reduction in tryptamine levels (at 0.3, 2, and 13 days, respectively).^18^ The *Lactobacillus* species *E. faecalis* specifically produces the neurotransmitter tyramine through tyrosine decarboxylation.^36^ No association of tyrosine decarboxylase with other members of the flora has been found, nor has any protein with significant homology to *E. faecalis. E. faecalis* has been treated with phage, and the tyrosine content was found to be reduced by 4 times at 0.3 days, 2.7 times at 2 days, and 4 times at 13 days.^18^ The *E. faecalis* strain from Atp4aSl/Sl mouse feces was isolated and used to isolate *E. faecalis* phages. The *E. faecalis* phages Efmus1, Efmus2, Efmus3 and Efmus4 were isolated from untreated raw sewage water. Three or four different phages targeting cytolytic *E. faecalis* (10^10^ PFUs) were administered to mice via gavage. It was found that the phages could significantly reduce the number of *Enterococcus* and levels of lysocytin.^37^ Phages may change the gene composition of their target hosts through the lysogenic pathway, affecting the expression of metabolite-related genes and later resulting in an increase or decrease in the levels of metabolites.

## (V) phages have a cascade of interactions with other bacteria

After reducing the number of their target bacteria, phages will also affect nontarget bacterial species in the symbiotic bacterial community of the intestinal tract through a cascade effect with the result that the overall number of intestinal bacteria is basically stable. For example, *E. coli* promotes the growth of *B. fragilis* and inhibits the growth of *Bacteroides vulgatus* through an interaction network. *E. coli* depletion by phage T4 resulted in growth inhibition of *B. fragilis* and enhanced growth of *B. vulgatu*s.^18^ In addition, after continuous application of phages to mice, the degree of intestinal microbiome dissimilarity between each mouse was gradually reduced. This finding indirectly suggests that phage predation may contribute to the stability of bacterial communities.

**Figure 1.**
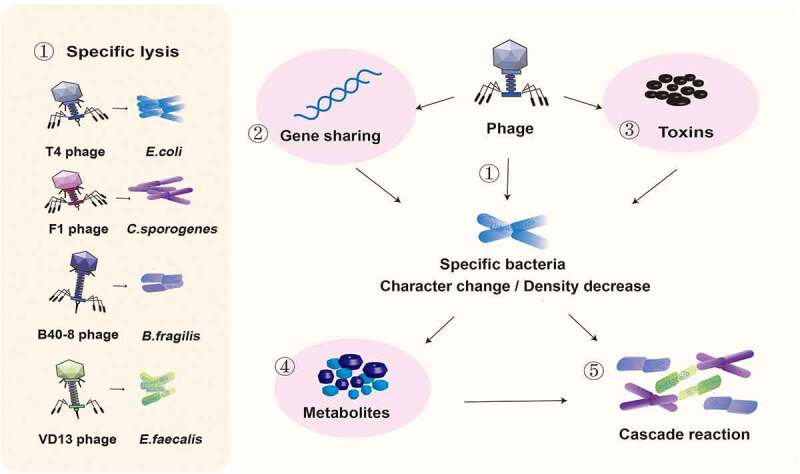
Effects of bacteriophage inoculation on the intestinal flora. ① Phages T4, F1, B40-8, and VD13 lysed only their susceptible bacteria *E. coli, C. sporogenes, B. fragilis* and *E. faecalis*. Phages directly affect susceptible bacteria through ① specific lysis, ② horizontal gene transfer and ③ encoding virulence genes, resulting in changes in intestinal bacteria and a decrease in their number. ④ The type of metabolites secreted may change due to the change in bacterial characteristics, and the total amount of metabolites secreted may decrease due to the decrease in bacterial number. ⑤ Changes in the abundance of bacteria and their metabolites can affect the intestinal environment and the growth of surrounding bacteria.

The effects of bacteriophage inoculation on the intestinal flora are shown in Figure 1.

### 2.2 Antagonistic mechanisms of intestinal bacteria against phage invasion

Humans are an important part of the Earth’s ecological environment. The intestine is a human organ directly connected with the external environment. Microorganisms that live in the environment also live in human intestines and perform similar functions. Bacteria and phages coexist in a complex and structured interaction network in the intestine, as in the environment.^38,39^

## (I) bacteria prevent phage adsorption by changing or hiding receptors

Phages infect their target bacteria with receptor-binding proteins (RBPs) on the surface of the bacteria. RBPs belong to different biochemical families, mainly represented by surface proteins, polysaccharides and lipopolysaccharides (LPSs). Bacteria prevent phages from binding to receptors by regulating receptor expression, mutating receptors and hiding receptors. For example, *V. cholerae* reduces O1 antigen expression by regulating the expression of manA and wbeL, two variable genes required for O1 antigen biosynthesis, which helps it avoid phage adsorption.^40^ The *E. coli* F strain can produce the outer membrane protein (OMP) TraT and prevents phage adsorption by masking or modifying the OmpA conformation.^41^ The capsule of the *Staphylococcus simulans* strain inhibits phage U16 from binding to its receptor, thus inhibiting phage U16 adsorption. Phages bind to *Pseudomonas* by polysaccharides.^42^ To prevent infection, *Pseudomonas* selects for mutations at many common sites associated with mucoid transformation, including mucA and algU, and inhibiting mucoidy.^43^ However, phages are also not static and can change their structure to bind to new receptors. The RBP of λ phage is encoded by the J gene and can bind to the host surface receptor LamB. When the expression of the LamB gene is suppressed, phages complete subsequent infection by changing the terminal structure of protein J and binding to the new receptor protein OmpF.^44^ Small modifications can also disguise receptors from phages. The *E. coli* K1 capsule can block phage T7 infection,^45^ and *Pseudomonas aeruginosa* O antigen modification and type IV pilus glycosylation can block phage infection.^46,47^ However, when *E. coli* produces a capsule to mask its LPS receptor, phage H4489A often encodes an extracellular hyaluronic acid lyase to degrade the capsule, thereby aiding adsorption.^48^ In addition, bacteria can also prevent phage adsorption by producing an extracellular matrix and through competitive inhibition. *E. coli* and *V. cholerae* reduce phage infection by providing phage-sensitive receptors on outer membrane vesicles (OMVs).^49,50^ *E. coli* FhuA is an iron transporter and an entry port for T5 phage. The antimicrobial molecule Microcin J25 uses FhuA as its receptor and competes with phage T5 for binding sites,^51^ resulting in a reduced chance of infection success.

## (II) bacteria destroy phage DNA through restrictive modification, inhibiting the integration and replication of phage genes

Restrictive modification in bacteria means that bacteria mark their genetic material by methylation at specific sites in the genome, and thus, unlabeled DNA is recognized, cleaved and degraded by a host of endonucleases. Type I, II and III restriction-modification (R-M) systems have methyltransferases and restriction endonucleases, which can protect the host by cutting DNA that is not recognized by its distinct code recognition.^52^ The defense island system associated with restriction-modification (DISARM) methylase, a widespread bacterial defense system, modifies the host CCWGG motif (W = A or T) as a self-DNA marker.^53^ The SspABCD-SspE phosphorothioate (PT) system in *Vibrio cyclitrophicus, E. coli* and *Streptomyces yokosukanensis* constitutes a defensive barrier against a diverse array of phages. SspABCD provides single-chain and high-frequency PTs. SspB in SspABCD binds to SspE as a nickel enzyme. SspE senses sequence-specific PTs by virtue of its PT-stimulated NTPase activity to exert its antiphage activity, and SspE inhibits phage propagation by introducing nicking damage to impair phage DNA replication.^54^ BREX (for bacteriophage exclusion) is a superfamily of common defense systems of bacteria, such as *Bacillus subtilis*. Bacteria differentiate self DNA from nonself DNA by methylating specific asymmetric sites using the BrxX (PglX) methyltransferase.^55,56^ Phage DNA that is not methylated will be unable to replicate and integrate with bacterial DNA. In the case of type I R–M systems, phages encode proteins that bind to the restriction complex and prevent its binding to unmodified recognition sites in phage DNA. A prototypical protein of this kind is OCR (overcome classical restriction) the product of the lytic bacteriophage T7 gene 0.3. As a DNA mimic, OCR binds many host proteins that interact with nucleic acids (e.g., DNA-dependent RNA polymerase). Goldfarb T et al. found that OCR overcomes *E. coli* BREX defense by specifically binding BrxX methyltransferase.^56^

## (III) bacteria degrade phage DNA by the CRISPR-cas system

The natural clustered regularly interspaced short palindromic repeats (CRISPR) system is divided into three phases: adaptation, expression and interference. During the adaptation phase, foreign DNA fragments (approximately 30–45 nucleotides, also known as prospacers) from invading plasmids or viruses are incorporated into the CRISPR sequence as new spacers. Selection of native spacers from foreign DNA is based on native prospacer adjacent motifs (PAMs). The new spacer provides memory for specific sequences to defend against the corresponding invading plasmid or virus. At the expression stage, the CRISPR array is transcribed into pre-CRISPR RNA, which is further processed into mature CRISPR RNA (crRNA). Each crRNA contains a conserved repeat sequence and a transcription interval that is complementary to the foreign DNA. A crRNA library can target multiple genetic elements because each crRNA corresponds to an invading sequence. In the interference phase, crRNA acts as a guide for specifically targeting the PAM, and Cas9 cleaves the matched DNA. Phage DNA is inserted into the CRISPR gene cluster to form a new spacer sequence, which is transcribed into crRNA. When the phage invades again, crRNA acts as a recognition marker to guide the Cas protein to the phage DNA and then acts as an endonuclease to degrade the phage DNA. For example, the Serrella III-A CRISPR-Cas complex can block phage infection, and Cas13 from the leptin type VI system can shear phage MS2 RNA in *E. col*i.^57^ However, five different “CRISPR-resistant” genes have been found in the genomes of phages infecting *P. aeruginosa*. Mutations in the CRISPR resistance gene of phages prevent them from infecting bacteria carrying a functional CRISPR/Cas system.^58^ *V. cholerae* serogroup O1 phage can encode part of the gene sequence of its own functional CRISPR-Cas system, which can target the functional CRISPR-Cas system at the initial stage of infection and destroy the host antiphage defense system. ICP1_2011_A phage targeted the *V. cholerae* O1 El Tor strain (harboring phage inducible chromosomal islands-like element 1 (PLE1)) by ICP1. The ICP1 phage CRISPR/Cas system consists of six Cas genes and two CRISPR loci (CR1 and CR2). Genomic organization of PLE1 targeted the CRISPR/Cas system of ICP1-related phage. Eleven ICP1-related phages from stools of cholera patients were isolated, five of which encode a CRISPR/Cas system located between ORF 87 and ORF 88 of the ancestral ICP1 genome14. The GC content of this CRISPR/Cas system is the same (~37%) as that of the rest of the ICP1 genome.^59^

## (IV) bacteria prevent the reinjection of phage DNA through a hyperinfection immune system

Superinfection exclusion (SIE) is a process in which proteins that may be anchored to the membrane or associated with elements on the membrane come into play when host bacteria are infected by one phage and another similar phage is adsorbed on the surface of the host cell, thus preventing secondary infections caused by similar phages. Arguijo-Hernández ES et al. presented genetic and biochemical evidence that the *E. coli* mEp167 Cor protein is an OMP. Cor interacts with OMPs, including OmpA, OmpC, OmpF and OmpW. Cor excluded fHUa-dependent phages in the lysogenic phage strain of *E. coli* mEp167.^60^ The superinfection exclusion A (SieA) system of *Salmonella typhimurium* carrying lysogenic phage *P22* can prevent infection by phage LMG178.^61^ When a bacterial cell is infected with a T-even phage, the phage-encoded proteins Imm and Sp emerge rapidly. Imm prevents DNA transfer across plasma membranes and partially inhibits the release of DNA from reinfected virions, while Sp inhibits the local degradation of bacterial wall proteins by phage-associated lysozyme.^62^

## (V) bacterial infection through the abi system leads to failure of phage lysis

The abortive infection (Abi) system is a mechanism by which cell death is induced after infection but before phage reproduction has completed, thereby protecting uninfected neighbors in a population. One of the Abi systems is the Rex system. *E. coli* phage λ of RexAB is a typical Abi system. The Rex system can be activated by the phage’s protein‒DNA complex. When the Rex system is triggered, the RexA protein activates the RexB protein, forming membrane channels that result in ATP leakage, loss of membrane potential, and obstruction of phage excretion.^63,64^ The Abi system can lead to the death of infected cells and often does not produce mature virus particles. Although this system has been studied for decades, we still do not fully understand its mode of action because of the complexity and diversity of the Abi system. There is also another Abi system called the toxin-antitoxin (TA) mechanism. This mechanism is based on the interaction between a toxin and an antitoxin. Endoribonuclease toxin-N was activated in *E. coli* after infection with T4 and other bacteriophages. Endoribonuclease toxin-N blocks phage development by cleaving viral mRNA and inhibiting its translation.^65^ The toxin targets essential cellular processes, causing the bacteria to hibernate or die at the same time. For example, for the lncL plasmid of *Klebsiella pneumoniae* carrying the PemIK (PemK/PemI) Type II TA System, overexpression of PemK toxin results in bacterial dormancy.^66^ To bypass the toxin, phages can encode antitoxins.^67,68^ Antitoxins can compete for the same binding site of toxins to disable their function. Alternatively, antitoxins can be chosen to neutralize the toxicity of toxins. Phages can generate a variety of escape methods by recombining to acquire host genetic material, and recombination can also facilitate phage acquisition of antitoxins.

**Figure 2.**
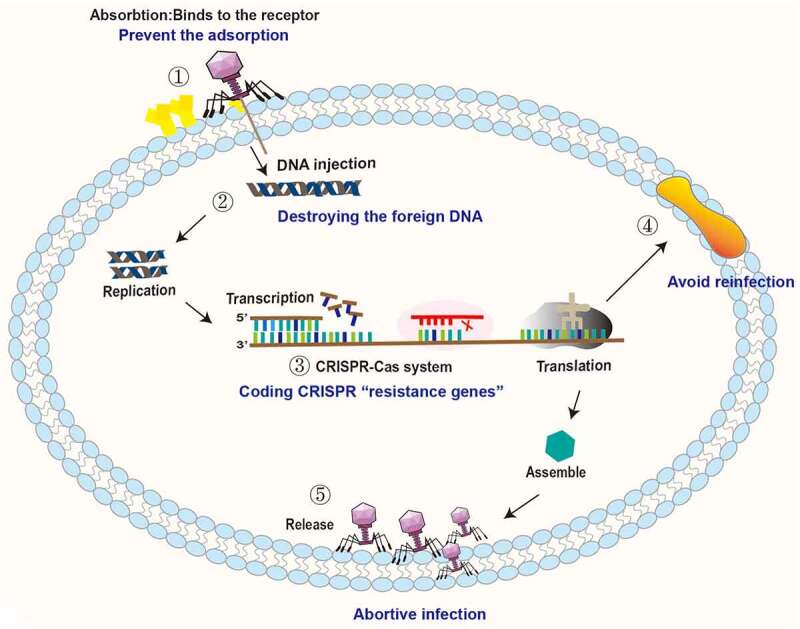
Antagonistic mechanisms of intestinal bacteria against phage invasion. ① Bacteria can prevent phage adsorption by changing and hiding receptors. ② Bacteria destroy the DNA of the invading phage through the restrictive modification-methylation pathway and inhibit the replication and integration of phage genes. ③ Bacteria degrade phage DNA by the CRISPR-Cas system. ④ Bacteria prevent reinfection by encoding superinfection immune system proteins. ⑤ Bacteria interfere with phage adsorption, injection, replication, assembly and release through the Abi system, leading to failure of phage lysis.

The antagonistic mechanisms of intestinal bacteria against phage invasion are shown in Figure 2.

### 2.3 Phages and bacteria coexist continuously in the gut

**Figure 3.**
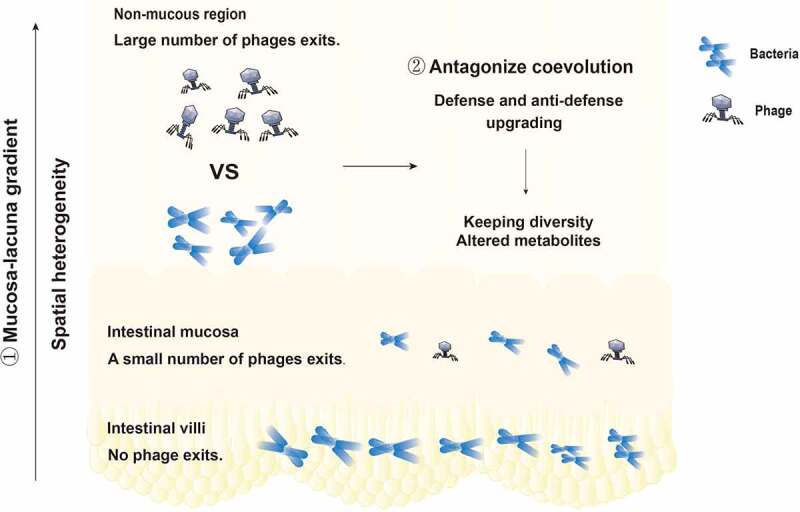
The cause of the persistent coexistence of phages and bacteria in the intestinal tract. ① The distribution of bacteriophages and bacteria is spatially heterogeneous. There are no phages in the intestinal villi, few phages in the intestinal mucosa, and a large number of phages in the intestinal lumen. The phage density in the intestinal tract presents a mucosal-lumenal gradient, providing a place for bacteria to avoid phage attack. ② The specific interaction between phages and bacteria, the competition between defense and anti-defense systems, and the way in which genes are shared to form new mutations or new species promote coevolution.

Phages are core members and potential regulators of the intestinal microbiota and play a vital role in maintaining the structure and function of the intestinal microbial community.^11^ Recent studies have shown that administration of virulent phages can strongly affect intestinal colonization of their target bacteria but still support long-term coexistence.^69^ The long-term coexistence of phages and gut bacteria is worth considering. Phages drive and maintain the stability and diversity of intestinal microecology by maintaining coevolutionary interactions with their microbial prey. Survival competition,^70–73^ gene transfer,^74^ and other factors are involved in the formation and turnover of microbial communities. Specific interactions between phages and bacteria, competition between defense and antidefense, and the mechanisms through which genes are shared to form new mutations and new species combinatorially promote coevolution. The replication and survival of bacteriophages rely on the bacterial host. A high density of bacteria is conducive to the survival of bacteriophages.^75^ Based on this, the “density dependence” hypothesis states that the more bacteria there are, the more phages there are. Two competing theories about the survival of viruses and bacteria are interesting. One is the kill-the-winner model, and the other is the piggyback-the-winner model.^76^ When bacterial density increases in an ecosystem, bacteria are called winners. As bacterial density increases in an ecosystem, so does the number of phages that infect those bacteria. It is widely believed that this growing population of phages then kills an increasing number of bacteria, limiting the population size. This is the model called “kill-the-winner”. The “piggyback-the-winner” model refers to the hypothesis that as potential host bacteria become increasingly numerous, some viruses forgo rapid multiplication and instead choose to remain steady in their host. These viruses multiply more slowly, avoid competing with other viruses, and avoid coming into contact with the host’s own immune defenses. These two seemingly contradictory theories are in fact how phages survive. Moreover, the latest research proposes a theory called bacterial spatial distribution, which suggests that the structured environment – the intestinal villi – can function as a spatial refuge for bacteria, allowing them to escape phage predation. This leads to the coexistence of bacteria and phages.^77^ Bacteriophages have specificity in killing intestinal symbiotic bacteria.^75^ In the intestinal microecosystem, bacteriophages and symbiotic bacteria have killing specificity, and there are also some bacteriophages and symbiotic bacteria that cannot interact with each other. The cause of the persistent coexistence of phages and bacteria in the intestinal tract is shown in Figure 3.

### 3. Intestinal phages induce diseases by regulating specific bacterial metabolism and initiating the immune response

## (I) phages regulate colitis via the immune response

Intestinal microbes are closely related to intestinal diseases such as colitis. Norman JM et al. (2015) observed a decrease in intestinal bacterial population diversity in patients with Crohn’s disease and ulcerative colitis (UC) and found that the occurrence of disease was associated with significant expansion of *Caudovirales* phages.^78^ Phage-lysed bacteria release bacterial surface molecules and intercellular contents to activate Toll-like receptor (TLR) signaling of the immune system or influence immune responses by regulating the bacterial community and inducing intestinal inflammation.^79^ Liu L et al. (2019) found that symbiotic viruses, such as bacteriophages, were recognized by RIG-I receptors expressed in intestinal antigen-presenting cells (APCs). RIG-I receptors promoted the production of IL-15 through the MAVS-IRF-1 signaling pathway and enhanced the activity and function of CD8ααα+TCR-αβ+ and CD8αβ+TCR-αβ+ intraepithelial lymphocytes (IELs). Mice lacking common viruses or MAVS are more likely to develop dextran sulfate sodium (DSS)-induced colitis, and restoring IELs in these mice through IL-15 supplementation reduces susceptibility to DSS.^80^ Gogokhia L et al. (2019) showed that *Lactobacillus* phage, *E. coli* phage, and *Bacteroidetes* phages stimulated interferon-gamma (IFN-γ) production through the nucleotide sensing receptor TLR9. Internalized phages triggered TLR-9 signaling with phage epitopes presented to CD4 + T cells, leading to the production of IFN-γ and the activation of inflammatory responses in the gut. Phages exacerbate colitis through TLR9 and IFN-γ. Before fecal microbiota transfer (FMT) and 4 weeks after FMT, Gogokhia L et al. performed total nucleic acid sequencing of 20 individuals with active UC. They found that patients who had a clinical response to FMT had a lower relative abundance of *Caudovirales* bacteriophages than patients who did not respond to therapy. Mucosal IFN-γ levels were positively correlated with phage levels, and phages in patients with active UC induced more IFN-γ than in healthy individuals.^69^ Adiliaghdam F et al. found that bacteriophages enriched from non-inflammatory bowel disease (IBD) individuals could actively elicit atypical anti-inflammatory innate immune programs. Bacteriophages enriched from IBD individuals divergently provoke proinflammatory macrophage responses. Increasing the ratio of non-IBD-associated bacteriophages in feces could partially restore the ability of macrophages to produce anti-inflammatory cytokines such as IL-22. Harnessing bacteriophages might offer therapeutic potential for IBD.^81^

## (II) Phages affect colorectal cancer by changing the bacterial community structure and regulating the immune microenvironment

Nakatsu G et al. (2018) performed a shotgun metagenomic analysis of stool samples from 74 colorectal cancer (CRC) patients and 92 non-CRC patients in Hong Kong and found that enterovirus population disorder was associated with early and advanced CRC.^82^ Zheng DW et al. (2019) observed a harmful overpopulation of *Fusobacterium nucleatum* in mice and patients with CRC, inhibiting the proliferation of beneficial *Clostridium butyricu*m.^83^ Phages infecting gram-negative bacterial hosts, such as enterotoxigenic *B. fragilis, E. coli* and *Clostridium nucleobacter*, are associated with CRC.^84–87^

Hannigan et al. (2018) analyzed 16S rRNA gene, whole shotgun metagenomic, and purified virus metagenomic sequencing of fecal samples from 30 healthy people, 30 patients with adenoma and 30 patients with CRC. The results showed that the cancer-associated virome consisted primarily of temperate bacteriophages that can indirectly induce cancer development by regulating the bacterial community composition.^88^ Moreover, phages can spread throughout sterile regions of bodies, including the blood, lymph, organs, and even the brain. Nguyen S et al. reported the rapid and directional transcytosis of diverse bacteriophages across confluent cell layers originating from the gut by incubating phage T4 with T84 (colon epithelial) cells and CaCo2 (colon epithelial) cells. Bacteriophages can access both the vesicular and cytosolic compartments of the eukaryotic cell, and transcytosed phages can traffic through the Golgi apparatus via the endomembrane system.^89,90^

There are cancer-promoting bacteria and cancer-inhibiting bacteria in the intestinal tract. Similarly, there may be cancer-promoting phages and cancer-inhibiting phages in the intestinal tract. The host adaptive immune response is active against phages and the gut microbiota, with certain microbial antigens able to stimulate memory T cells. Some intestinal microbial antigens can stimulate memory T cells. At the same time, intestinal microorganisms that cross-react with tumor-associated antigens can activate CD4+ and CD8 + T cells that specifically secrete IFN-γ, contributing to the antitumor immune response. Flukiger et al. found that the symbiotic bacterium *Enterococcus hirae* contained a prephage, which encoded an MHC class I restricted antigen in its tape measure protein (TMP). Mice colonized by *E. coli* containing a prephage can induce a TMP-specific CD8 + T-cell reaction after cyclophosphamide treatment. Furthermore, the expression of the mimic carcinogenic peptide PSMB4 in phage TMP1 can effectively inhibit the growth of tumors to improve the effect of immunotherapy.^91,92^ Murgas P et al. (2018) demonstrated that single-chain DNA containing M13 phage had high immunogenicity and could specifically target tumor cell surfaces, trigger inflammation and invasion of activated innate immune cells, overcome tumor-related immunosuppression, and promote antitumor immunity.^91^ Dong X et al. (2020) achieved the specific elimination of *Clostridium* symbiosis by electrostatic assembly of silver nanoparticles (AgNPs) on the surface capsid protein (M13@Ag) of M13 phage that specifically binds *Clostridium*. M13 phage activated the host immune system and inhibited CRC development by activating APCs to alter the tumor immune microenvironment and reduce myeloid-derived suppressor cell (MDSC) amplification at the tumor site.^93^

## (III) phages induce alcoholic liver disease development by increasing bacterial exotoxin release

Alcoholic hepatitis is the most serious form of alcohol-related liver disease (ALD), with a mortality rate of up to 40%. Lu Jiang et al. (2020) extracted VLPs from the feces of 89 patients with alcoholic hepatitis included in a multicenter observational study and conducted metagenomic sequencing to characterize *enteroviruses*. The authors observed that the diversity of viral groups in fecal samples of patients with alcoholic hepatitis was most significantly higher. The abundances of *Escherichia, Enterobacteria* and *Enterococcus* phage were abnormally high, and the number of mammalian viruses, such as *Parvoviridae* and *Herpesviridae*, was significantly increased. Duan Y et al. (2019) compared patients with alcoholic hepatitis, subjects with alcohol use disorders, and healthy controls and found a more than 2,700-fold increase in the number of *E. faecalis* in the gut microflora of the alcoholic hepatitis group.^37^ Thirty percent of *E. faecalis* strains have genes that encode an exotoxin called cytolysin. The presence of lysin-positive (lysozyme) *E. faecalis* was associated with liver disease severity and mortality in patients with alcoholic hepatitis: 89% of lysin-positive patients died within 180 days of admission, compared with 3.8% of those who were lysin-negative. Next, the researchers used a mouse model of ALD to demonstrate that cytolysin directly contributes to disease progression. Cytolysins directly induce liver cell death in vitro and promote liver damage in ethanol-induced liver disease (chronic alcohol diet) mice. Mice colonized with *E. faecalis* and then fed ethanol had more severe liver damage and hepatic steatosis than the control group. The same trend in ALD severity was observed in germ-free mice receiving fecal transplants from cytolysin-positive patients with alcoholic hepatitis. Researchers have developed a novel treatment that uses phages to specifically target *E. faecalis*. Four phages capable of lysing cytolytic *E. faecalis* from Atp4aSl/Sl mouse feces were isolated from sewage. Intragastrically administered with these phages, Atp4aSl/Sl mice had less severe liver damage, steatosis, and inflammation following chronic intragastric administration of ethanol than those administered control phages or vectors. Importantly, according to 16S rRNA sequencing analysis, the fecal abundance of *E. faecalis* was reduced after the phage intervention. According to qPCR analysis, cytolysin levels were reduced.^94–96^

## (IV) phages may alleviate graft-versus-host disease by reducing inflammation and regulating immunity

Graft-versus-host disease (GVHD) is the most common serious complication after hematopoietic cell transplantation. It is caused by an immune attack by T cells contained in the graft, which recognize the recipient’s foreign tissue, activate lymphocytes, and then develop an immune response to the graft cells. Recent data indicate a correlation between the degree of intestinal microbiome imbalance and outcome and mortality. *Enterobacter, Staphylococcaceae, Actinomycetes* and *Firmicutes* are related to the occurrence and severity of GVHD.^97–99^ Promising preliminary results were obtained for the treatment of GVHD using FMT, in which bacterial-deficient fecal extracts effectively mediated the beneficial effects of FMT; successful FMT is associated with an increase in the number of cauda viruses, suggesting that phages may be a key part of the microbiota responsible for the efficacy of FMT.^100,101^ Studies have shown that phages can protect intestinal epithelial cells from bacterial invasion and directly inhibit inflammation by interacting with epithelial cells. For example, phages can inhibit reactive oxygen species (ROS) production induced by *E. coli* in human neutrophils and monocytes and endotoxin-induced ROS production.^102,103^ Phage tail filament proteins bind to host cell surface-specific receptors and attach to the cell surface. Different phages have different receptors on the host cell surface. LPS is a common receptor for phages such as T3, T4 and T7. LPS is an important bacterial factor inducing the production of inflammatory cytokines, disrupting the internal environment. Phages can interfere with LPS release and induce mononuclear cells to produce IL-10 and IL-1 receptor antagonists to reduce inflammation^104–106^ and can regulate the functions of dendritic cells, monocytes and granulocytes, inhibit the NF-kappaB pathway stimulated by LPS, and regulate immune function.^107^ Miernikiewicz P et al. found that the infiltration of leukocytes into the lungs, liver and spleen was markedly increased in mice treated with LPS compared to that in mice treated with PBS. The LPS structure was associated with its ability to induce an inflammatory response. Binding of phage protein gp12 to the hydrophilic core disturbed its function in the formation of TLR4-MD-2-LPS complexes that could lead to immune stimulation.^108^ Phages reduce the production of inflammatory mediators, which may reduce the development of GVHD and have a protective effect against GVHD in the clinic.

## (V) phages and specific bacteria affect rheumatoid arthritis through the immune pathway

Rheumatoid arthritis (RA) is a highly inherited multifactorial autoimmune disease. Studies have shown that RA may be related to changes in the intestinal flora, and intestinal microbes may be involved in immune regulation in RA development as inflammatory mediators.^109,110^ RA is characterized by the presence of antibodies against cyclic citrullinated protein (CCP) in serum antibody-positive individuals. The heritability of RA is estimated at 40–60%. Familial risk was significantly increased in first-degree relatives (FDRs) of patients diagnosed with RA. Mangalea et al. performed intestinal phage analysis of FDRs of RA patients (with and without anti-CCP antibodies) and found that the intestinal phages of the FDR (high RA risk) group were enriched in those targeting *Bacteroidaceae*. The number of *Streptococcaceae* phage and *Lachnospiraceae* phage in the CCP+FDR group was increased.^111^ Phages use bacteria such as *Lachnospiraceae* as their target hosts, and their proliferation is related to an increased abundance of bacteria such as *Lachnospiraceae* in the CCP+ group.^112^ Phages enriched in the CCP+FDR group carry the accessory metabolic gene (AMG) PHNP, which encodes a phosphodiesterase and regulates phosphate degradation.^108^ Mangalea also found that phages enriched in the CCP+FDR group carried clusters of transferases such as mannose-phosphotransferase (algA, xanB, rfbA, wbpW, and PSLB), mannose-heptanose transferase (gmhC, hldE, waaE, and rfaE), GALE (epimerase) and glm transaminase.^111^ Phages of the CCP+FDR group may influence the formation of bacterial cell wall polysaccharides and biofilms through transferases,^113^ thus participating in immune evasion. Phages also have immunomodulatory effects through their inherent anti-inflammatory properties and can directly regulate lymphocytes by translocation to multiple tissues.^104^ The intestinal phage community composition of patients with RA can fluctuate with changes in immune system function and disease, and this community composition has potential as a biomarker for early disease detection.^114^

## (VI) phages affect blood glucose homeostasis by altering intestinal bacterial composition and metabolism

Type 1 diabetes mellitus (T1DM) is a disease in which the autoimmune system attacks Langerhans insulin-producing cells. Type 2 diabetes mellitus (T2DM) is a chronic metabolic disease with high blood sugar in the context of insulin resistance and impaired insulin secretion. The development of both T1DM and T2DM is associated with intestinal microbiome disorders. The abundance of *Bacteroidetes* increased in T1DM, while the abundance of butyric-producing bacteria decreased.^115^ In T2DM, the abundance of sulfate-reducing bacteria and Enterobacteriaceae increased, while the abundance of *Firmicutes* and butyric-producing bacteria decreased.^116–118^ Changes in the intestinal microbiome composition can affect the functions of the microbiome, such as increased membrane transport of sugars or branched amino acids, increased enzyme activities involved in exogenous or carbohydrate metabolism, and decreased functions involved in cell motility and butyric acid synthesis.^116,117^ Such changes also affect the metabolic functions of the microbiome, such as short-chain fatty acids (SCFA) production and vitamin metabolism, which are negatively correlated with insulin resistance.^119^ The binding of SCFAs to GPR43 and GPR41 increases plasma glucagon-like peptide-1 (GLP-1) and peptide YY levels and improves glucose homeostasis.^120^ Phages may be involved in the occurrence and development of diabetes by affecting bacterial hosts.^121^ For example, prophage activation significantly induced *E. coli* biofilms to release amyloid. Amyloid-derived curly fibers may trigger T1DM progression through the TLR2-MyD88-NF-kB signaling pathway.^122^ Enhancement of intestinal gram-negative bacterial lysis by phages releases LPS, leading to systemic subclinical inflammation and affecting insulin sensitivity.^123,124^ Zhao G et al. observed changes in enteric phages over time, changes in enteric viruses preceding autoimmunity, and disease-related viral phages associated with specific components of the bacterial microbiome.^125^ This finding suggests that phages may cause dysglycemia by altering the immune regulation of the gut microbiome. In addition, the biological properties of phages give them the ability to regulate host abundance, thereby influencing bacterial community structure through a cascade of positive and negative interactions between bacterial components of the intestinal microbial community. When the cecal virus population was transferred from lean mice to obese mice, transgraft-induced transgenic changes in the fecal virus group resulted in weight fluctuations in obese mice, and blood glucose parameters returned to normal.^126^ Transplanting the fecal microbiota of a healthy lean donor to metabolic disease subjects has been shown to improve peripheral insulin sensitivity, with 65% of subjects experiencing a 10% increase in glucose loss during the first 6 weeks after treatment, and bacteriophage groups that could explain these differences were identified and examined.^127^

**Figure 4.**
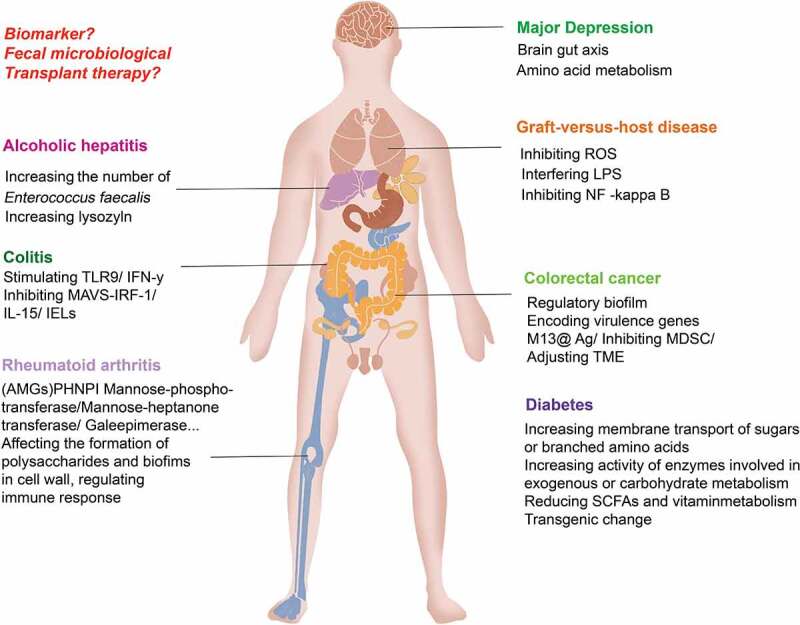
Intestinal phages induce the development of diseases by regulating and increasing the release of bacterial exotoxins, regulating bacterial community metabolism, and initiating immune responses. Changes in intestinal phages, bacteria and their metabolites through fecal microbiota transfer may help alleviate diseases. Changes in bacterial metabolites closely related to disease may be able to be used as markers for disease surveillance.

The mechanism of intestinal phage-induced disease is shown in Figure 4.

## 4. Application of phage in the treatment of intestinal diseases

With the emergence of bacteria resistant to many antibiotics, there is increasing interest in phage therapy. Phages are host-specific and can target specific pathogenic bacteria without directly affecting the normal flora of the host. Phages are easy to grow and purify; phages are “live drugs” that increase in number as the target bacterial population spreads and can be administered in small doses; phages are not toxic, and they attack only bacteria, not human cells; and phages are self-limiting, and once the target bacterial population is eliminated, the number of phages will greatly decrease. Therefore, the use of phage therapy is currently a hot topic.

## (I) phage cocktails as antimicrobial therapy

The problem of rising antibiotic resistance in recent years has revived researchers’ interest in phage therapy, as phage are the natural enemies of bacteria. The most common bacterial infections of the gastrointestinal tract are diarrhea caused by *Clostridioides difficile*, dysentery caused by *Shigella*, gastroenteritis caused by strains of *E. coli*, cholera caused by *V. cholerae*, and salmonellosis caused by *Salmonella enteritidis*. Many studies have assessed the potential of phages for treating gastrointestinal infections caused by *E. coli, Enterobacte*r,^128^ *V. cholera*e,^129^ and *C. difficil*e.^130^ Phage therapy has successfully provided support for the treatment of intestinal infection in diabetic foot infection,^131^ lung infection,^132^ corneal abscess^133^ and other conditions. For example, the Phagoburn (phase I–II clinical trial) project funded by the European Union under the 7th R&D Framework Programme has conducted a large-scale clinical trial of lysophages for the treatment of infected burn wounds.^134^ Nestle (Switzerland), in collaboration with The International Centre for Diarrheal Disease Research Dhaka Hospital, Bangladesh, has conducted a phase I/II trial to evaluate the safety and efficacy of oral T4-like phage cocktails in hospitalized children with acute bacterial diarrhea,^135^ further facilitating the study of phages in enteric infectious diseases. Because of the synergy between individual phages, a phage cocktail may be more effective than a single phage. A 68-year-old man with necrotizing pancreatitis with multidrug-resistant *Acinetobacter baumannii* infection failed to improve after multiple rounds of antibiotic therapy. The researchers isolated *A. baumannii* from the patient and screened phages in two different laboratories, mixing the phages targeting the bacteria to produce a phage cocktail, and rapid improvement was achieved with the administration of the phage cocktail by intraperitoneal catheter and intravenous injection.^136^ Phage injection can be an effective method for controlling antibiotic-resistant bacteria in treating bacterial infections, by using phages that target specific bacteria.

## (II) phage vaccines enhance specific immune responses

Phages are composed mainly of nucleic acids and proteins wrapped in a capsid that protects them from nuclease degradation. Phages are excellent carriers of DNA owing to their ability to maintain stability over a range of pH values and resist nuclease degradation. In eukaryotic hosts, phages are inert granular antigens that cannot trigger pathogenesis. Therefore, in recent years, many studies have explored the use of phages as nanodrug platforms to develop vaccines. At present, most phage vaccines are phage display vaccines and phage DNA vaccines.^137^ Phage display vaccines insert the DNA sequence of the reference foreign protein or polypeptide into the appropriate location of the phage coat protein structural gene so that the foreign gene is expressed along with the coat protein expression. The phage then captures specific molecules such as replicases, pathogen viral factors, or bacterial cell antigens using antigen-binding peptides displayed on the surface of coat proteins. Phage display technology can be used to prepare anti-intestinal pathogenic microorganism vaccines, which can assist in the diagnosis and treatment of intestinal infectious diseases. Phage DNA vaccines are entire phage particles that are used as vectors for genes encoding protective antigenic peptides that are carried to target cells to produce antigens. The proportion of deoxycytidylinate-phosphodeoxyguanosine (CpG) sequences in the phage genome is relatively high, and TLR-9 can recognize CpG sequences and initiate an immune response.^138,139^ DNA nanodevice vaccines, precisely assembled with two types of molecular adjuvants and an antigenic peptide, have been identified to induce a powerful antigen-specific T-cell response to yield tumor regression, as well as a long-term T-cell response to protect the body from tumor recurrence.^140^ Phage DNA vaccines targeting intestinal tumors to initiate adaptive immunity while improving the intestinal microenvironment are worthy of further exploration as potential treatments.

## (III) phage module exchange or gene editing

Bacteria are central to human health and disease conditions, but existing tools for editing microbial consortia are limited. For example, broad-spectrum antibiotics do not offer precise control over bacterial communities. Synthetic biology is beginning to solve this problem; microbial synthetic biology refers to a customized biological application system that modifies the internal structure of microorganisms to mimic the functions of specific engineering systems.^141^ Using synthetic biology to edit phages can enlarge their host range, enhance the ability of phages to penetrate biofilms, and improve the targeted use of phages in antibacterial therapy. Ando H et al. directed *E. coli* phage scaffolds to pathogenic *Yersinia* and *Klebsiella*; in contrast, *Klebsiella* phage scaffolds were directed to *E. coli* through a module exchange of phage tail components. Synthetic phages can effectively kill target bacteria and selectively remove specific bacteria from multispecies bacterial communities using mixtures based on common viral scaffolds.^142^ Phages modified by the CRISPR/Cas system can sensitize resistant bacteria by eliminating drug-resistant plasmids. Synthetic biologist Timothy Lu of Massachusetts Institute of Technology and his team used DNA programming CRISPR technology to build engineered phages that specifically infect and kill drug-resistant bacteria. The phage targets bacteria with drug-resistant DNA sequences via fragments of RNA. If the bacterium contains a drug-resistant DNA sequence, the RNA can bind to this sequence and bind the Cas9 enzyme to cleave the bacterial DNA and kill the bacterium.^143^ Jalasvuori et al. showed that the use of plasmid-dependent phages on *E. coli* and *Salmonella enterica* resulted in a significant reduction in the number of resistant bacteria.^144^ Bacteriophages are potential candidates for replacing antibiotics in the control of infectious bacteria. However, host bacterial resistance to phages is inevitable. Interestingly, gut bacteria that respond to phage resistance recover their sensitivity to certain antibiotics. *P. aeruginosa* has a receptor-binding site, the outer membrane porin M (OprM), of the multidrug efflux systems MexAB and MexXY. Chan BK et al. found that the evolution of *P. aeruginosa* resistance to phage attack changed the efflux pump mechanism, causing increased sensitivity to antibiotic drugs from ciprofloxacin and tetracycline.^145^ Fong K et al. found that sensitivity to tetracycline was increased, while *S. enterica* developed resistance to Bacteriophage SI1 via mutated genes involved in type VI secretion that contributes to LPS production.^146^

## (IV) phage-targeted delivery of therapeutic drugs for intestinal diseases

Phages can be engineered to form self-assembled nanomaterials with affinity properties. Drugs attached to the surface of the phage can be carried by the phage to a specific location in the body. Drug-carrying phages can then improve a disease environment by specifically lysing bacteria, releasing drugs or binding to antibody receptors on the membranes of specific cancer cells, participating in endocytosis, or treating intracellular degradation. For example, dextran nanoparticles loaded with the cancer chemotherapeutic irinotecan covalently attached to azide-modified phages have been used to treat the overgrowth of *F. nucleatum*, which was significantly inhibited in mice with in situ-induced CRC or naturally formed CRC after oral or intravenous administration of the modified phages, and CRC was significantly ameliorated under the therapeutic effect of phage-delivered irinotecan. It was also found that oral phage-guided irinotecan nanoparticles did not cause significant changes in blood cell count, immunoglobulin and histamine levels, or liver and kidney function in piglets.^83^ The emergence of this type of technology for covalent modification of viral particles lays a foundation for the transformation of viral capsids into targeted drug carrier systems.^147^ Kovacs et al. were able to coat genome-free MS2 capsids with polyethylene glycol (PEG) chains and incorporate 50–70 copies of fluorescent drug mimics into the capsids. Studies have shown that despite extensive modification, the capsid remains assembled, making it an effective delivery vessel for drugs.^148^ Drugs can also attach to the phage surface without damaging the cell target. For example, drug-carrying liposomes can deliver drugs to cells via phage-liposome complexes.^149^ Phage-carried drugs naturally existing in the intestinal tract have the characteristics of excellent targeting, strong therapeutic effects and few toxic side effects, holding the potential for serving as ideal drug delivery tools.

**Figure 5.**
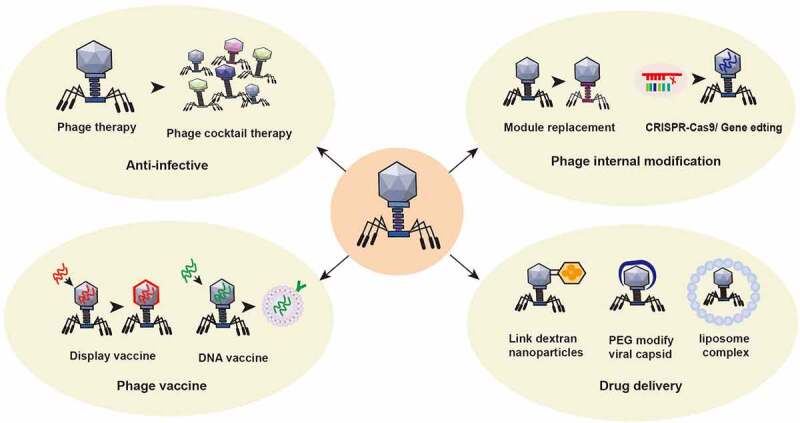
Application of phage in the treatment of intestinal diseases. ① Using the nature of phage, a phage cocktail is made to target bacterial infection. ② The structure of phages can be modified by switching phage modules or by applying CRISPR gene editing technology to change the intestinal bacterial host or enhance its antagonism to the intestinal bacterial host. ③ Preparation of phage display vaccines and phage DNA vaccines to enhance specific immune responses. ④Using phage to treat specific intestinal or other bacteria with targeted delivery of disease treatment drugs. For example, glucan nanoparticles are covalently linked to azide-modified phages, polyethylene glycol capsid-modified phages, phage-liposome complexes and other anti-colorectal cancer drugs.

The application of phages in the treatment of intestinal diseases is shown in Figure 5.

## (V) potential limitations of phage therapy

Not all phages are suitable for therapeutic use. Lytic phages are commonly used for clinical treatment. It is required to have specific fracture characteristics and stable fracture effects (independent of temperature and environment) and to ensure safety and efficiency (no toxin protein gene in the genome). Bacteriophage preparations may contain endotoxic proteins of host bacteria, and the bacterial endotoxins released after lysing the host may affect the normal function of the body. Phages that do not lyse bacteria quickly are less effective against them. Phages usually act only on a certain genus or species of bacteria, and some even act only on a limited number of strains of a species. Phage preparations mainly include phage nucleic acids, capsid proteins and so on in the clinic. These preparations are more complex than common clinical pharmaceutical preparations with a single chemical structure. It is difficult to evaluate the activity and purity of drugs, and it is impossible to accurately define the method of administration, dosage form, dose, concentration and administration time of phage preparations. The pharmacokinetics of phage preparations are not clear, and the safety of phage after entering the body cannot be evaluated. Phages are proliferating, evolving, and gene-editing organisms that interact with the body’s immune system.^150–154^ It is not clear whether the use of phages can also adversely affect the human immune system.
